# Transcriptome analysis reveals the mechanisms for mycorrhiza-enhanced salt tolerance in rice

**DOI:** 10.3389/fpls.2022.1072171

**Published:** 2022-12-19

**Authors:** Chen Hsieh, Yun-Hsin Chen, Kai-Chieh Chang, Shu-Yi Yang

**Affiliations:** ^1^ Department of Horticulture and Landscape Architecture, National Taiwan University, Taipei, Taiwan; ^2^ Institute of Plant Biology, National Taiwan University, Taipei, Taiwan

**Keywords:** Arbuscular mycorrhizal symbiosis, salinity, transcriptome, *Oryza sativa L.*, Pi homeostasis, cell wall

## Abstract

More than half of the global population relies on rice as a staple food, but salinization of soil presents a great threat to rice cultivation. Although previous studies have addressed the possible benefits of arbuscular mycorrhizal (AM) symbiosis for rice under salinity stress, the underlying molecular mechanisms are still unclear. In this study, we found that mycorrhizal rice had better shoot and reproductive growth and a significantly higher K^+^/Na^+^ ratio in the shoot. The reactive oxygen species (ROS) scavenging capacity in rice shoots was also improved by AM symbiosis. To elucidate the molecular mechanisms required for AM-improved salt tolerance, transcriptome analysis revealing the differentially expressed genes (DEGs) based on the response to AM symbiosis, salinity or specific tissue was performed. Thirteen percent of DEGs showed tissue-preferred responses to both AM symbiosis and salt stress and might be the key genes contributing to AM-enhanced salt tolerance. Gene Ontology (GO) enrichment analysis identified GO terms specifically appearing in this category, including cell wall, oxidoreductase activity, reproduction and ester-related terms. Interestingly, GO terms related to phosphate (Pi) homeostasis were also found, suggesting the possible role of the Pi-related signaling pathway involved in AM-enhanced salt tolerance. Intriguingly, under nonsaline conditions, AM symbiosis influenced the expression of these genes in a similar way as salinity, especially in the shoots. Overall, our results indicate that AM symbiosis may possibly use a multipronged approach to influence gene expression in a way similar to salinity, and this modification could help plants be prepared for salt stress.

## Introduction

Rice (*Oryza sativa L.*), belonging to the family Gramineae (Poaceae), is a major food crop for more than half of the global population and is very susceptible to saline soil, which is one of the most important obstacles to crop production worldwide (Flowers and Yeo, 1995; Porcel et al., 2012). In view of the evidence from a recent study, up to 50% of cultivated land will be degraded by 2050 due to salinization (Hossain, 2019). Sodium (Na^+^) is the main toxic ion in salinized soil (Wakeel, 2013). Many cytosolic enzyme activities are activated by potassium but inhibited by sodium (Flowers et al., 1977). Salt stress can also reduce photosynthesis efficiency and induce changes in cell wall properties, ionic toxicity (primary effect) and osmotic stress (secondary effect), leading to the accumulation of reactive oxygen species (ROS) (Shomer et al., 2003; Sudhir and Murthy, 2004; Sharma et al., 2012; Singh et al., 2014). To increase tolerance to stress, plants have evolved a variety of physiological and biochemical mechanisms in response to salt damage. Regarding cell wall modification, previous studies have reported that monocot and dicot expansins play positive roles in plant resistance to salt stress (Yan et al., 2014; Li et al., 2015; Jadamba et al., 2020). To remove excess ROS, plants have developed a system including nonenzymatic and enzymatic antioxidants (Ahmad et al., 2010; Das and Roychoudhury, 2014). To mitigate the damage of toxic ions, mediating roles of transporters in Na^+^ absorption from root to shoot and Na^+^ compartmentalization within leaf tissues and cells, including Na^+^/H^+^ antiporter SOS1 (salt overly sensitive), Na^+^/K^+^ transporter HKT (high affinity potassium transporter) and vacuolar Na^+^/H^+^ antiporter NHX (sodium/hydrogen exchanger), are critical for salinity tolerance (Horie et al., 2007; Olias et al., 2009; Keisham et al., 2018). The role of osmoprotectants and hormones in salt stress tolerance has also been discussed (Gupta and Huang, 2014)

Arbuscular mycorrhizal (AM) fungi can form mutualistic symbiotic relationships with more than 80% of terrestrial plant species and 90% of agricultural plants (Smith and Read, 2010). These symbiotic relationships can enhance plant nutrient uptake from the soil, thereby improving growth and stress tolerance (Porras-Soriano et al., 2009; Kapoor et al., 2013; Rivero et al., 2018; Begum et al., 2019). AM fungi increase photosynthetic efficiency, secondary metabolite production, antioxidant activity and accessibility of water and nutrients to the plant and maintain ion balance under salt stress (Porcel et al., 2012; Evelin and Kapoor, 2014; Sarwat et al., 2016; Evelin et al., 2019). Moreover, AM symbiosis helps to prevent excessive Na^+^ uptake and transport from roots to shoots, enhancing the absorption of essential cations, such as K^+^, Ca^2+^, and Mg^2+^, and increasing the K^+^/Na^+^ or Mg^2+^/Na^+^ ratio in host plants under saline conditions (Giri et al., 2003; Giri and Mukerji, 2004; Colla et al., 2008; Evelin et al., 2019). AM-enhanced salt stress tolerance of rice has also been reported (Porcel et al., 2015; Porcel et al., 2016; Tisarum et al., 2020). The AM fungus *Claroideoglomus etunicatum* enhances the quantum yield of the rice plant (*O. sativa* L. cv. puntal) for photosystem II and decreases nonphotochemical quenching (NPQ) under salt stress, thereby improving the carbon dioxide (CO_2_) fixation efficiency (Porcel et al., 2015). Moreover, upland rice plants inoculated with AM fungi (*Glomus etunicatum*) have a higher level of photosynthetic abilities, photosynthetic pigment, stomatal conductance, transpiration rate, osmolyte production (e.g., total soluble sugar and free proline), osmotic potential and grain yield under salt stress (Tisarum et al., 2020). Furthermore, the expression of rice transporter genes (*OsSOS1*, *OsNHX3*, *OsHKT2;1* and *OsHKT1;5*), which are involved in vacuolar sodium sequestration and Na^+^ recirculation from shoots to roots, is upregulated in AM-colonized plants in a saline environment (Porcel et al., 2016). These studies all report the positive effects of AM symbiosis on salt stress tolerance in rice using phenomic approaches (Porcel et al., 2015; Porcel et al., 2016; Tisarum et al., 2020). Even though ample evidence has demonstrated that AM symbiosis has positive effects on plant physiological responses under salinity stresses, the underlying mechanism of how AM symbiosis manipulates its host plant to manage salt stress is still limited. A more recent RNA-seq-based transcriptome analysis revealed that the positive effects of AM symbiosis at the transcriptional level of *Sesbania cannabina* alleviate salt stress, which is mainly enriched in photosynthesis, ROS scavenging and specific transcription factors (Ren et al., 2019). Transcriptome analysis revealed that AM-induced genes in the roots of *Casuarina glauca* under salinity were enriched in antioxidant enzyme activity, carbohydrate metabolism, cell wall and ion transport (Wang et al., 2021). However, such comprehensive transcriptome analysis to reveal how AM symbiosis alleviates salt damage in rice has not yet been performed.

In this work, we investigated the effect of AM symbiosis on the growth and ion homeostasis of rice plants under salt stress. By using transcriptome analysis, we focused on differentially expressed genes (DEGs) showing tissue-preferred responses to both AM symbiosis and salt stress to illustrate how the transcriptome regulation of mycorrhizal rice plants responds to salt stress.

## Materials and methods

### Plant materials and growth conditions

Seeds of rice (*O. sativa L. japonica* cv. Nipponbare) were sterilized and germinated on 1/2 Murashige and Skoog (MS) medium with 0.8% agar. After one week, rice seedlings were transferred and grown in plastic tubes containing sterilized sands without (mock) or with sand inoculum containing spores of *Rhizophagus irregularis* (Ri, purchased from *Mycorise^®^ASP*, *Premier Tech*, Rivière-du-Loup, Québec, Canada). Plants were grown in a phytochamber with a 12-h day/night cycle at 30/28°C and 70% air humidity. The plants were regularly watered for the first week after inoculation and fertilized every second day with one-half Hoagland solution containing 25 μM phosphate (Pi). At five weeks postinoculation (5 wpi), mock and mycorrhizal plants were divided into two batches, in which one batch was treated with fertilizer solution supplemented with 150 mM NaCl (saline condition) and the other batch was grown under nonsaline conditions. At 8 wpi, shoots and roots were collected and separated to detect the physiological, biochemical and molecular responses. One biological replicate was the combination of two plants, and three biological replicates were collected from each treatment.

### Mycorrhizal quantification

Root samples were stained with Trypan blue, and mycorrhizal colonization was quantified with a modified gridline intersection procedure as described (Paszkowski et al., 2006).

### Plant phenotyping

For biomass measurement, the dry weight was measured after two days at 70°C in a hot-air oven. To monitor the tissue ion content, 0.05 g of chopped dry samples was digested with 2 ml 65% HNO_3_ and 0.5 ml H_2_O_2_ (both Suprapur; Merck) in a MarsXpress microwave digestion system (CEM, Matthews, NC, USA). After that, the samples were diluted with Milli-Q water (Millipore Co., MA, USA) to 20 ml, filtered using a 0.45‐μm membrane filter and injected into an inductively coupled plasma (ICP) analyzer. The elemental profile of plant samples was determined using inductively coupled plasma‐optical emission spectrometry (ICP‐OES; PerkinElmer OPTIMA 5300) according to a previously described method with some modification (Shanmugam et al., 2011). The relative ion concentration (mg/L) of each sample was obtained based on the wavelength intensity and calibration standard curve. For 3,3’-diaminobenzidine (DAB) staining, the youngest fully expanded leaves detached from 8-week-old plants were soaked in DAB solution (0.1%) overnight and then decolorized in bleaching solution (96% ethanol:acetic acid:glycerol = 3:1:1) at 70°C until the brown spots appeared clearly. To statistically present the H_2_O_2_ content visualization by DAB staining, all pictures were first transformed into 8-bit grayscale images. The percentage of brown area (the area of brown spots divided by the area of whole leaf) was quantified by using ImageJ.

### RNA extraction, cDNA synthesis, RT−PCR and RT−qPCR

RNA extraction, cDNA synthesis, RT−PCR, and RT−qPCR were performed as previously reported (Gutjahr et al., 2008). Total RNA was extracted from 100 mg shoot or root tissue by using TRIzol Reagent (Invitrogen™) following the manufacturer’s instructions. For the following gene expression analysis, genomic DNA was removed from total RNA using DNase I (RNase-free, Invitrogen™). After DNase I treatment, purified RNA samples were reverse transcribed using the Moloney murine leukemia virus reverse transcriptase kit (Invitrogen™) with oligo (dT) primers for cDNA synthesis. The RT-qPCRs were performed with SYBR^®^ Green Supermix (2X) (Bio-Rad) on a CFX Connect Real-Time PCR Detection System (Applied Biosystems) as described in the manufacturer’s protocol. Transcript levels were normalized to constitutively expressed *Cyclophilin2* (Gutjahr et al., 2008). The RT−qPCR primers were designed using Primer3 web version 4.1.0 (http://primer3.ut.ee/). All RT−qPCR primers are listed in **Supplementary Table S9**.

### Illumina library preparation, sequencing and functional annotation

A total amount of RNA (≥ 4 μg) per sample was used for transcriptome sequencing. The quantity and purity of the purified RNA was assessed using a NanoDrop ND-2000 (Thermo Scientific, Wilmington, MA, USA) and an Agilent 2100 Bioanalyzer (Agilent Technologies, Inc., Santa Clara, CA, USA) before Illumina NGS sequencing. To construct the sequencing libraries, only high-quality RNA samples were used (OD260/280 = 1.8~2.0, OD260/230 ≥ 2.0, RIN ≥ 7.0). After purification, end repair, and ligation to sequencing adapters, strand-specific sequencing libraries were constructed by using the Illumina HiSeq 4000 platform (2 × 150 bp paired end) and following the handbook for the NEBNext^®^ Ultra™ RNA Library Prep Kit for Illumina^®^ (Genomics Biotechnology Co., Ltd, Taipei, Taiwan). The read depth is 6 Gb. The quality control of raw reads was evaluated using FastQC software v0.11.8 (http://www.bioinformatics.babraham.ac.uk/projects/fastqc/). The low-quality reads (Q-value < 20) and adaptors were removed using the sequence preprocessing tool Trimmomatic v0.39 (Bolger et al., 2014). After low-quality reads and adaptors were removed, all reads were mapped to *Oryza sativa* v7.0 from Phytozome 13 (Goodstein et al., 2012) with STAR (Dobin et al., 2013) and quantified with Salmon (Patro et al., 2017) using the nf-core/rnaseq pipeline (Ewels et al., 2020).

### Differentially expressed gene analysis

Genes with low expression (log2CPM < -3) were filtered out based on the CPM value. DEGs were identified using the edgeR (empirical analysis of DGE in R) package v3.10.5 by comparing the counts per million (CPM) values between treatment groups (Robinson et al., 2010). Three treatment groups (salt, AM and tissue) were categorized to identify DEGs responding to salt, AM symbiosis or tissue from pairwise comparison between samples with one factor difference. For example, salt-responsive genes were identified between the same tissues with the same mycorrhizal treatment but grown under control and salinity conditions. AM-responsive genes were identified between the same tissues with the same salinity treatment but grown under mock and mycorrhizal conditions. Tissue-responsive genes were identified between roots and shoots with the same salinity and mycorrhizal treatment. In each group, four pairwise comparisons were performed. An adjusted p value and false discovery rate (FDR) < 0.05 and an absolute value of log2-fold-change > 1 were considered significant thresholds. To further analyze the overlap of DEGs among different treatment groups, a custom Python script was used to plot the Venn diagram of the DEGs responsive to salt, AM symbiosis and tissue.

### GO enrichment analysis

After obtaining the DEGs in each region from Venn diagram analysis, a gene ontology (GO) enrichment analysis of the DEGs was performed with CARMO, a web-based tool for GO analysis (Wang et al., 2015) with “MSU RGAP ID” selected. The output GO list from CARMO was submitted to REVIGO (Supek et al., 2011) for visualization for interpretation. The bubble plot in the manuscript was plotted with a custom Python script using the scatterplot function from the package seaborn (Waskom et al., 2017). The GO terms from each region were sorted by FDR and filtered based on FDR < 0.05. Only the top 15 terms were visualized on the bubble plot.

### Gene expression heatmap visualization

Fold change values were calculated by edgeR (Robinson et al., 2010). TPM values were calculated by Salmon (Patro et al., 2017). Custom Python scripts were used to visualize the fold change value of the selected genes with the clustermap function from the seaborn package (Waskom et al., 2017).

### Statistical analysis

The experiment to observe phenotypic data was arranged with three to seven biological replicates in each treatment. The mean values were compared using two-way ANOVA followed by a least significant differences (LSD) *post hoc* test and analyzed by R software.

### Accession numbers

Sequence data from this article can be found in the Michigan State University Rice Genome Annotation Project database (http://rice.plantbiology.msu.edu) using the following accession numbers: *granule-bound starch synthase II* (*OsGBSSII*) (LOC_Os07g22930), *xyloglucan endotransglucosylase/hydrolases 19* (*OsXTH19*) (LOC_Os03g01800), *low phosphate root 5* (*OsLPR5*) (LOC_Os01g03640), *purple acid phosphatase 7* (*OsPAP7*) (LOC_Os11g34720) and *OsCyclophilin2* (LOC_Os02g02890). The sequencing files discussed in this publication have been deposited in NCBI’s Gene Expression Omnibus (Edgar et al., 2002) and are accessible through Gene Expression Omnibus (GEO) Series accession number GSE200863 (https://www.ncbi.nlm.nih.gov/geo/query/acc.cgi?acc=GSE200863).

## Results

### Phenotypic changes in mock and mycorrhizal plants under salt stress

**Figure 1:**
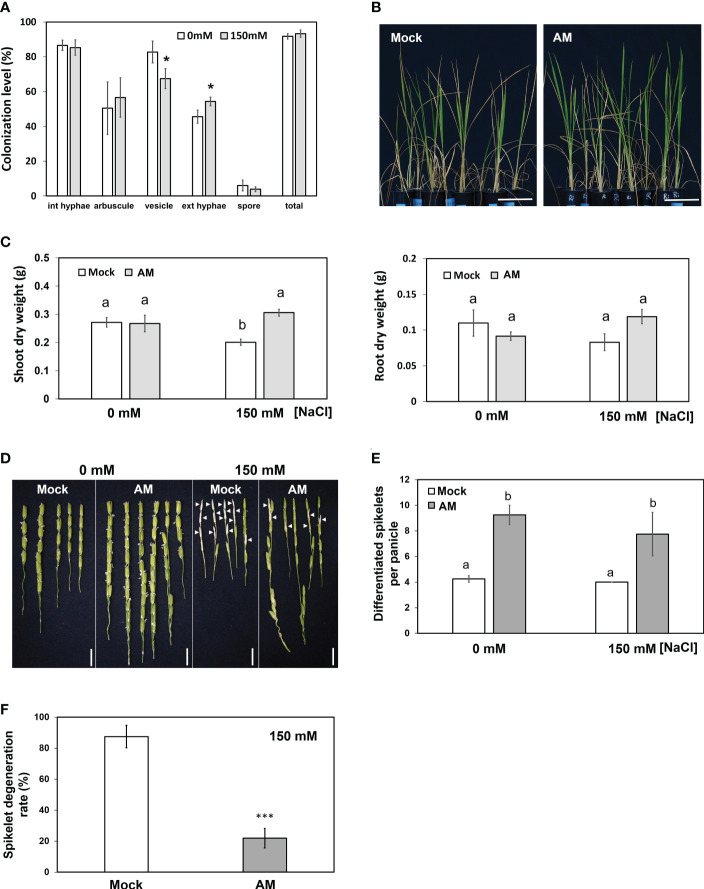
Physiological and agronomic traits of mock and mycorrhizal plants under salt stress. **(A)** Fungal colonization levels expressed as a percentage of colonized roots. **(B)** Phenotype of rice plants under salt stress. **(C)** Dry weight of shoots and roots. **(D)** Spikelet phenotype. Arrowheads indicate the typical salt-injured spikelets. **(E)** Comparison of spikelet number per panicle in mock and mycorrhizal plants. **(F)** Spikelet degeneration rate of mock and mycorrhizal plants under salt stress. The spikelet degeneration rate was defined as the degenerated spikelet number divided by the total number of spikelets per panicle. Rice plants were grown without (mock) or with the AM fungus *R. irregularis* (AM) for 5 weeks and then treated with normal (0 mM NaCl) or salt solution (150 mM NaCl) for 3 weeks. Standard error is derived from 3-7 biological replicates. One plant was considered one biological replicate in **(C-F)**, and two plants were considered one biological replicate in **(A)**. Different letters represent significant differences at *p* < 0.05 (two-way ANOVA followed by a least significant differences *post hoc* test). *p < 0.05, ***p < 0.001 for Student’s t test. Scale bar = 10 cm in **(B)** and 1 cm in **(D)**.

To examine whether mycorrhizal rice plants could maintain better growth under salt stress, 5-week-old rice seedlings inoculated with the AM fungus *R. irregularis* (Ri) or without (mock) were grown under nonsaline (0 mM NaCl) or saline (150 mM NaCl) conditions for another three weeks. To investigate the effect of salinity on fungal growth, the fungal colonization level was quantified. Mock plants did not show the presence of AM fungi under either saline or nonsaline conditions (data not shown). The average fungal colonization levels reached 91% and 93% under nonsaline and saline conditions, respectively, indicating that AM fungi successfully colonized rice roots. The abundance of vesicles was slightly reduced by salinity, and the level of extraradical hyphae was higher under salt stress. The levels of the remaining fungal structures were not significantly different between nonsaline and saline conditions (**Figure 1A**). These results indicated that salt stress had a mild impact on AM symbiosis.

Mycorrhizal plants showed fewer wilted blade tips than mock plants under saline conditions (**Figure 1B**). Under nonsaline conditions, both the shoot and root biomass of mock plants and mycorrhizal plants were not different (**Figure 1C**), reflecting the fact that rice is an AM-nonresponsive plant (Smith et al., 2004; Li et al., 2006; Hata et al., 2010; Smith and Read, 2010). On the other hand, under saline conditions, the shoot biomass of mock plants but not mycorrhizal plants was severely decreased by salt stress (**Figure 1C**). The dry weight of mycorrhizal plant shoots was significantly higher than that of the mock group (1.5-fold) under salt stress. In contrast, AM symbiosis did not influence root biomass significantly under either nonsaline or saline conditions (**Figure 1C**). These results suggested that AM symbiosis may help rice plants maintain better shoot growth under salt stress.

We then aimed to further examine whether AM symbiosis also had positive effects on reproductive growth (panicle development) under salt stress. Compared with the mock plants, the mycorrhizal plants showed a 2.17- and 1.93-fold increase in differentiated spikelet numbers under control and salt stress conditions, respectively (**Figure 1E**). In addition, AM-colonized plants contained fewer salt-injured spikelets (**Figure 1D**), and the spikelet degeneration rate (number of salt-injured spikelets divided by total number of spikelets) in mycorrhizal plants was significantly lower than that in mock plants under salt stress (**Figure 1F**). These results further indicated that AM symbiosis could enhance spikelet tolerance to salt stress.

To evaluate the effect of salt stress on nutrient content, the concentrations of Na^+^, K^+^ and P were measured. Under salt stress, the Na^+^ concentration significantly increased compared to nonsaline conditions in both mock and mycorrhizal plants. Strikingly, mycorrhizal shoots presented a lower concentration of Na^+^ than mock shoots under salt stress (**Supplementary Figure S1A**). The ability to maintain a high cytosolic K^+^/Na^+^ ratio is an indicator of plant salt tolerance (Gregorio and Senadhira, 1993; Wu et al., 2013), so the K^+^ concentration was also measured to calculate the K^+^/Na^+^ ratio. The only difference in K^+^ concentration between mock and mycorrhizal plants was found in shoot tissue under salt stress, in which mock shoots accumulated more K^+^ than mycorrhizal shoots (**Supplementary Figure S1B**). Salt stress significantly reduced the K^+^/Na^+^ ratio in both mock and mycorrhizal plants. In roots, the K^+^/Na^+^ ratio of mycorrhizal plants was significantly higher than that of mock plants under nonsaline conditions (**Supplementary Figure S1C**). However, in shoots, the K^+^/Na^+^ ratio of mycorrhizal plants was significantly higher than that of mock plants under saline conditions (**Supplementary Figure S1D**). Even though the shoot/root Na^+^ ratio was significantly increased by salt stress, the shoot/root Na^+^ ratio was significantly lower in mycorrhizal plants than in mock plants, suggesting that mycorrhizal plants restrict sodium movement from roots to shoots (**Supplementary Figure S1E**). Previous studies showed that phosphate enhanced plant growth under saline conditions (Okusanya and Fawole, 1985). Therefore, we also measured the phosphorus content of plants. The results showed that the phosphorus content in mycorrhizal plants significantly increased in both shoots and roots compared with mock plants under both nonsaline and saline conditions (**Supplementary Figure S1F**). The positive effect of AM symbiosis not only enhanced salt tolerance, as shown by the K^+^/Na^+^ ratio, but also increased the efficiency of phosphate uptake.

**Figure 2:**
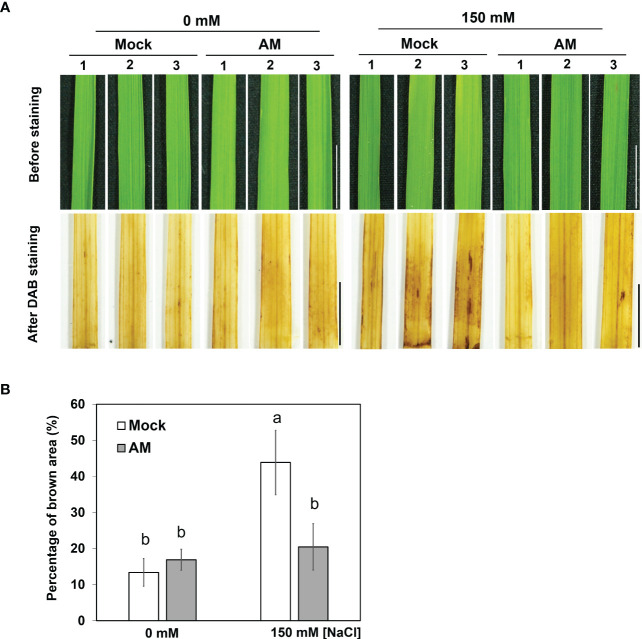
DAB staining in rice shoots under salt stress **(A)** Accumulation of H_2_O_2_ visualized in leaf blades by DAB staining after 3 weeks of salt stress. The eighth fully expanded leaves from 5-6 plants were detached and incubated in DAB solution for 16 hrs, and representative leaves from 3 plants are presented. Mock, nonmycorrhizal plants; AM, mycorrhizal plants inoculated with *R. irregularis*. Scale bar = 1 cm. **(B)** The percentage of brown area (the area of brown spots divided by the area of whole leaf) of DAB staining was quantified by using ImageJ. The standard error was derived from 5-6 biological replicates (one plant was considered one biological replicate). Different letters represent significant differences at *p* < 0.05 (two-way ANOVA followed by a least significant differences *post hoc* test).

Since AM symbiosis maintained shoot growth under salt stress (**Figure 1C**), whether the ROS level was different between mock and mycorrhizal shoots was further analyzed by visualizing H_2_O_2_ content histochemical stained with 3,3′-diaminobenzidine (DAB). It was obvious that the area of brown coloration was larger in mock shoots than in mycorrhizal shoots under salt stress (**Figure 2**). The results suggested that AM symbiosis may enhance the reduction in H_2_O_2_ levels in the shoots to protect the rice plant from oxidative damage under salt stress.

### Identification of differentially expressed genes regulated by AM symbiosis in response to salt stress

To better understand the salt tolerance mechanism in mycorrhizal plants at the molecular level, RNA sequencing was used to investigate differential gene expression in response to AM symbiosis under salt stress. RNA-seq data were generated from the roots and shoots of mock and mycorrhizal plants grown under nonsaline and saline conditions with two to three biological replicates. Twenty‐two paired‐end libraries (**Supplementary Table S1**) were generated, and 683,233,862 and 656,077,984 paired-end 150 bp raw reads were obtained from the control and salt treatment samples, respectively. After removing the low-quality raw reads (Q-value < 20) and trimming adaptor sequences, a total of 671,602,666 cleaned reads with > 90% Q30 bases from nonsaline samples and 635,393,116 from saline samples were selected as high-quality reads for further analysis (**Supplementary Table S1**). All the high-quality reads served as input to the nfcore/rnaseq pipeline. The genome reference of *Oryza sativa* v7.0 was downloaded from Phytozome 13 (https://phytozome-next.jgi.doe.gov/). Sample correlation matrix analysis revealed a strong similarity between the biological replicates (**Supplementary Figure S2**).

**Figure 3:**
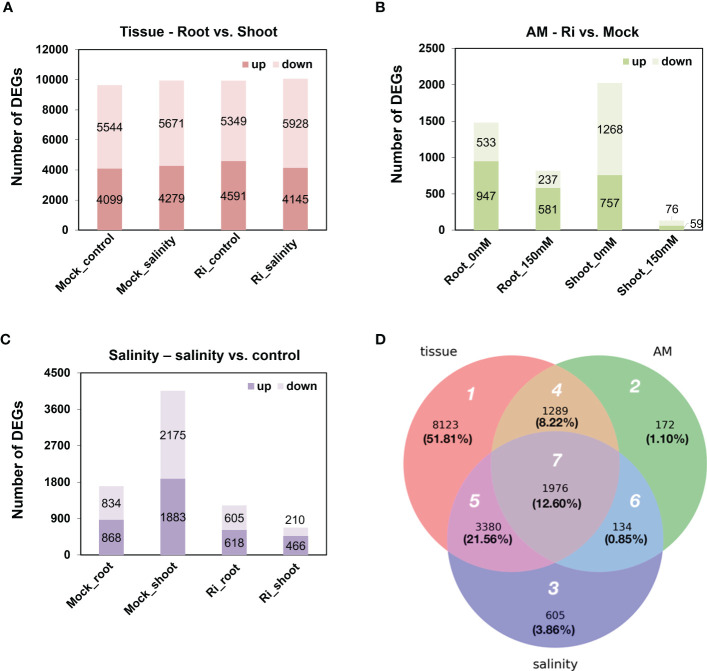
Differentially expressed genes (DEGs) regulated by tissue, AM symbiosis, and salinity stress **(A)** The number of up- and downregulated DEGs in roots compared to shoots. Upregulated referred to higher expression in roots compared to shoots and vice versa. **(B)** The number of up- and downregulated DEGs by AM symbiosis. Upregulated referred to higher expression in mycorrhizal (Ri) tissues than mock tissues and vice versa. **(C)** The number of up- and downregulated DEGs by salinity. Upregulated referred to higher expression in tissues grown under salinity conditions (150 mM) than under control conditions (0 mM) and vice versa. A total of 8 groups of samples were pairwise compared to identify DEGs regulated by tissue, AM symbiosis or salinity with cutoffs oflog_2_(fold-change)> 1 and FDR < 0.05. **(D)** Venn diagram showing the overlap of DEGs among the three treatment groups. White number: region number; black number: number of DEGs; black number inside parenthesis, percentage of the total DEGs.

Differentially expressed genes (DEGs) affected by one of the three factors, salinity, AM symbiosis and tissue, were identified with cutoffs of | log_2_ (fold-change) | > 1 and FDR < 0.05. For tissue-preferred expression, approximately nine thousand genes showed differential expression patterns between shoots and roots in each condition, and among these genes, approximately 45% showed higher expression in the roots than in the shoots. Moreover, this ratio was similar and not affected by salinity or AM symbiosis (**Figure 3A**). For AM-regulated expression, the number of DEGs regulated by AM symbiosis varied. For example, 2025 and 135 genes were regulated by AM symbiosis in the shoots grown under control and salinity conditions, respectively, and 1480 and 818 genes were regulated by AM symbiosis in the roots grown under control and salinity conditions, respectively. The number of AM-regulated DEGs was dramatically reduced in the roots and shoots grown under salt stress compared to those under control condition. Interestingly, more genes were downregulated in shoots but upregulated in roots by AM symbiosis under both control and salinity conditions (**Figure 3B**). For salinity-regulated expression, the number of DEGs regulated by salinity also varied. For example, 4058 and 676 genes were regulated by salinity in the shoots under mock and mycorrhizal conditions, respectively, and 1702 and 1223 genes were regulated by salinity in the roots under mock and mycorrhizal conditions, respectively. The number of salinity-regulated DEGs was significantly reduced in the tissues colonized by AM fungi, especially in shoots. In addition, salinity stress upregulated more genes in roots than no stress under both mock and mycorrhizal conditions. However, more genes were downregulated by salinity stress in mock shoots but upregulated in mycorrhizal shoots (**Figure 3C**). These results showed that the effect of AM symbiosis and salinity on gene expression was less pronounced under salinity and mycorrhizal conditions, respectively. In addition, compared to mock shoots, mycorrhizal shoots showed differential responses to salt stress.

In total, 15,679 DEGs were identified from the three treatment groups (**Figure 3A**). To further reveal the difference in DEGs among the three treatments, a Venn diagram was generated, and these DEGs were categorized into the seven regions (**Figure 3D**). In region 1, 8,123 genes (51.81%) were differentially expressed between shoots and roots and were not regulated by AM symbiosis or salinity treatment. In region 2, 172 genes (1.10%) were regulated by AM symbiosis but did not show tissue-preferred or salinity-responsive expression patterns. In region 3, 605 genes (3.86%) were regulated by salt stress but did not show tissue-preferred or AM-responsive expression patterns. In region 4, 1,289 genes (8.22%) showed AM-responsive and tissue-preferred expression patterns but did not respond to salinity treatment. In region 5, 3,380 genes (21.56%) showed salt-responsive and tissue-preferred expression patterns but did not respond to AM symbiosis. In region 6, 134 genes (0.85%) were regulated by both AM symbiosis and salinity but showed similar expression levels in both shoots and roots. In region 7, 1,976 genes (12.60%) were not only responsive to AM symbiosis and salt stress but also showed either shoot- or root-preferred expression patterns (**Figure 3D**).

### Gene ontology enrichment analysis showed the functional profiles of AM-regulated genes

**Figure 4:**
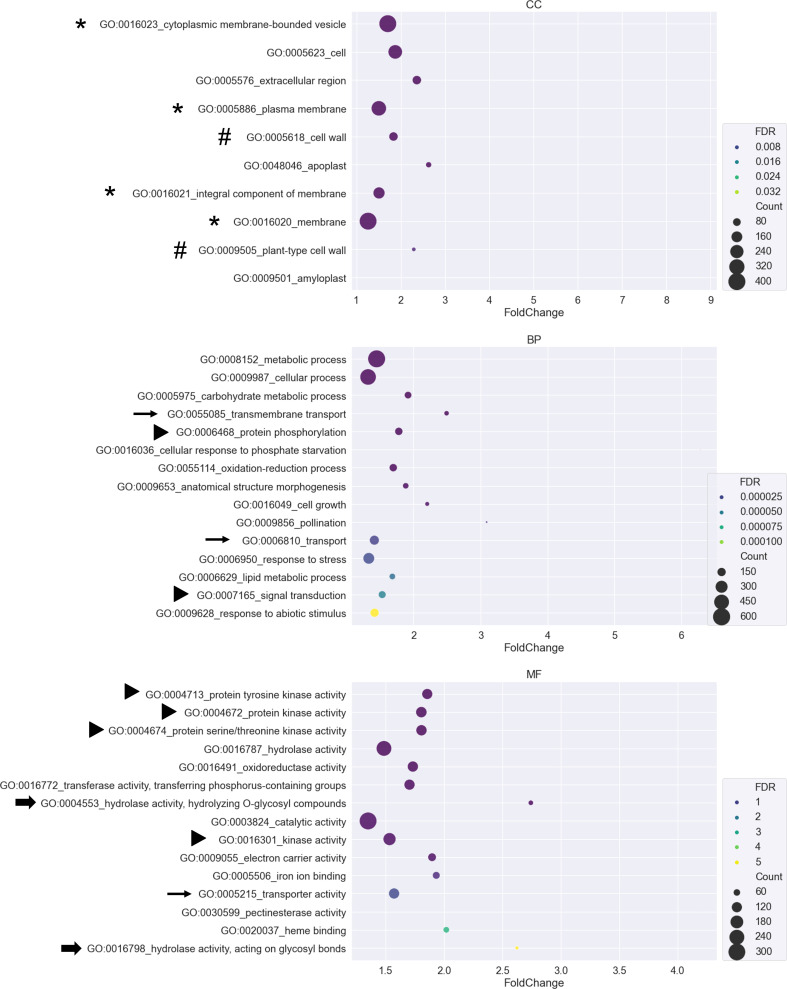
GO enrichment analysis for DEGs from region 7 A bubble plot was used to visualize the Gene Ontology (GO) enrichment analysis of DEGs belonging to region 7. GO terms from the cellular component (CC), biological process (BP), and molecular function (MF) categories were plotted. The X-axis represents the fold change of the GO enrichment level of the gene set over the background. The size of the bubbles represents the number of genes. The color of the bubbles represents the false discovery rate (FDR). GO terms with FDR greater than 0.05 were removed from the list. Only the 10-15 GO terms with the smallest FDR value were visualized in each category. Symbols in front of GO terms showed that these GO terms shared similar descriptions.

To identify the functional categories of each region, DEGs of each region in **Figure 3D** were submitted to CARMO for GO enrichment analysis. Enriched GO terms with FDR < 0.05 were retained for inspection and visualization. Only region 1, region 4, region 5, and region 7 obtained enriched GO terms that were significant (**Supplementary Table S2**). In region 1, genes showing tissue-preferred expression patterns were enriched in terms such as “thylakoid membrane organization”, “photosynthesis”, and “ATP binding” (**Supplementary Table S2**). In region 4, genes showing AM-responsive and tissue-preferred expression patterns were enriched in terms such as “biosynthetic process”, “lipid metabolic process” and “sequence-specific DNA binding transcription factor activity” (**Supplementary Table S2**). In region 5, genes showing salinity-responsive and tissue-preferred expression patterns were enriched in terms such as “secondary metabolic process”, “response to abiotic stimulus” and “electron carrier activity” (**Supplementary Table S2**). In region 7, genes showing tissue-preferred expression in response to both AM symbiosis and salinity might be important for AM-enhanced salt stress tolerance. Among the 10-15 GO terms with the smallest FDR value belonging to either the cellular component (CC), biological process (BP), or molecular function (MF) category, several GO terms contained similar descriptions. For example, four and two GO terms contained “membrane” and “cell wall”, respectively. Three and two GO terms contained “transport” and “hydrolase activity”, respectively. Six GO terms were related to signal transduction, including “protein tyrosine kinase activity” and “protein serine/threonine kinase activity”. In addition, the most enriched GO term is “cytoplasmic membrane-bounded vesicle” (**Supplementary Table S2** and **Figure 4**).

Since we are interested in how AM symbiosis enhanced salt stress tolerance, a 3-way Venn diagram of the enriched GO terms from region 4 (tissue-preferred and AM-responsive), region 5 (tissue-preferred and salinity-responsive), and region 7 (tissue-preferred and AM/salinity-responsive) was generated to find specific GO terms derived from each region (**Supplementary Figure S3** and **Table S3**). The GO terms that overlapped between the three regions (g7 region in **Supplementary Figure S3**) included more general terms such as “carbohydrate metabolic process”, “catalytic activity”, “cation binding”, “hydrolase activity”, “metabolic process”, and “transport”. Stress-related terms such as “response to abiotic stimulus” and “response to stress” and signaling-related terms such as “protein kinase activity”, “protein phosphorylation”, “protein serine/threonine kinase activity” and “protein tyrosine kinase activity” were also found (**Supplementary Table S3**). The GO terms that were specific to tissue-preferred and AM-responsive DEGs (g1 region in **Supplementary Figure S3**) included “ATPase activity”, “fatty acid biosynthetic process” and “transmembrane receptor protein serine/threonine kinase signaling pathway” (**Supplementary Table S3**). The GO terms that were specific to tissue-preferred and salinity-responsive DEGs (g2 region in **Supplementary Figure S3**) included transport-related terms such as “amino acid transport”, “divalent metal ion transport” and “sugar transmembrane transporter activity”. Secondary metabolite-related terms such as “secondary metabolic process” and “ent-kaurene synthase activity” and photosynthesis-related terms such as “photosynthesis, light harvesting” and “photosynthesis, light reaction” were also found. Interestingly, reproduction-related terms such as “pollen−pistil interaction”, “recognition of pollen” and “stamen development” were also identified (**Supplementary Table S3**). Finally, GO terms specific to DEGs representing tissue-preferred and AM/salinity-responsive expression patterns might be involved in AM-enhanced salt tolerance (g3 region in **Supplementary Figure S3**). In the g3 region, GO terms could be classified into several categories. One category was related to esters, such as “carboxylic ester hydrolase activity” and “hydrolase activity, acting on ester bonds”. Another category was related to reproduction, such as “pollen tube growth” and “pollination”. GO terms related to the cell wall, such as “cell wall modification”, “cellular glucan metabolic process”, “glucan biosynthetic process”, “pectinesterase activity” and “xyloglucan:xyloglucosyl transferase activity”, were also found. Intriguingly, many GO terms related to Pi homeostasis were found in the g3 region, such as “acid phosphatase activity”, “cellular phosphate ion homeostasis”, “cellular response to phosphate starvation”, “myo-inositol hexakisphosphate biosynthetic process”, “phosphate ion transport” and “positive regulation of cellular response to phosphate starvation” (**Supplementary Table S3**). The expression pattern of genes belonging to GO terms specific to the g3 region will be closely investigated in future studies.

### Genes related to cell wall modification, ester-related, reproduction and oxidoreductase activity are involved in the response to AM symbiosis and salt stress

**Figure 5:**
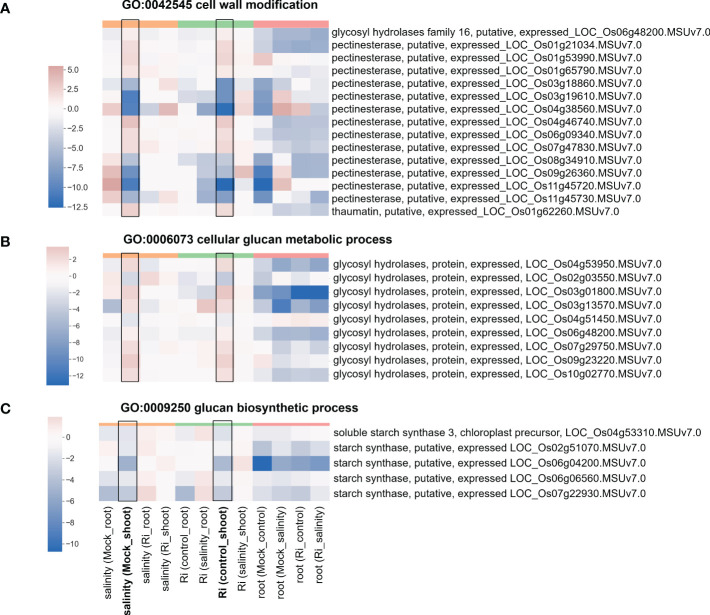
Heatmap showing the fold change of DEGs belonging to cell wall-related GO terms **(A)** Expression profile of DEGs from region 7 with the GO term “GO:0042545 cell wall modification”. **(B)** Expression profile of DEGs from region 7 with the GO term “GO:0006073 cellular glucan metabolic process”. **(C)** Expression profile of DEGs from region 7 with the GO term “GO:0009250 glucan biosynthetic process”. The log2(fold change) values of DEGs from the selected GO terms were visualized with a heatmap. The color bar on top of the heatmap indicates the DE comparison groups: orange – salinity effect; green – AM symbiosis; pink – tissue difference. On the x-axis, each column represents a pairwise comparison to show the effect causing the DE, and inside the parentheses are the condition.

**Figure 6:**
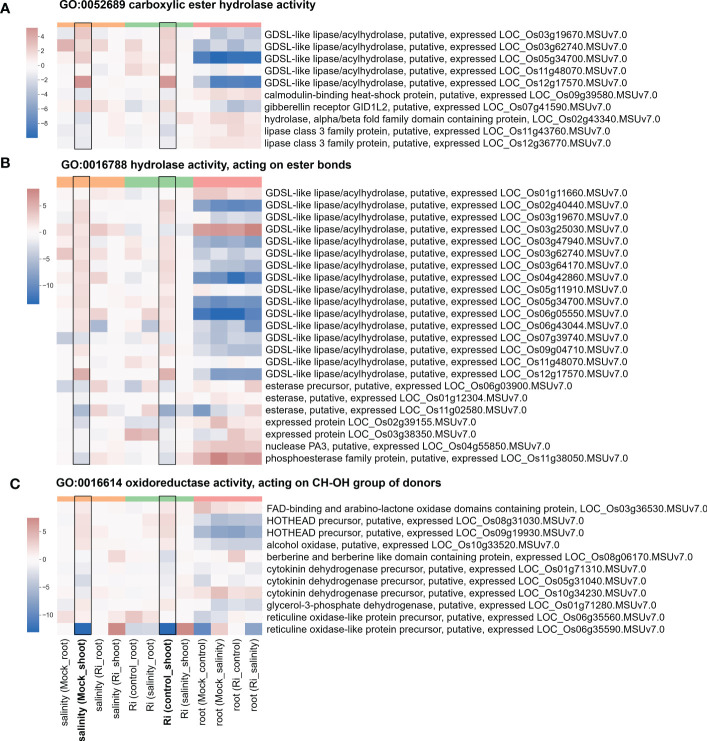
Heatmap showing the fold change of DEGs belonging to ester-related and oxidoreductase activity GO terms **(A)** Expression profile of DEGs from region 7 with the GO term “GO:0052689 carboxylic ester hydrolase activity”. **(B)** Expression profile of DEGs from region 7 with the GO term “GO:0016788 hydrolase activity, acting on ester bonds”. **(C)** Expression profile of DEGs from region 7 with the GO term “GO:0016614 oxidoreductase activity, acting on CH-OH group of donors”. The log2(fold change) values of DEGs from the selected GO terms were visualized with a heatmap. The color bar on top of the heatmap indicates the DE comparison groups: orange – salinity effect; green – AM symbiosis; pink – tissue difference. On the x-axis, each column represents a pairwise comparison to show the effect causing the DE, and inside the parentheses are the condition.

The expression of DEGs belonging to region 7-specific GO terms (g3 region) was visualized by heatmap. Most genes belonging to cell wall-related GO terms, such as “cell wall modification”, “cellular glucan metabolic process” and “glucan biosynthetic process”, showed higher expression in shoots than in roots (**Figure 5** and **Supplementary Table S4**), suggesting their possible critical role in the shoots. Interestingly, in the shoots, the expression patterns of these genes in response to salinity and AM symbiosis were similar. For example, pectinesterase (LOC_Os01g21034) was induced by salinity in the mock shoots (salinity (Mock_shoot) column) and upregulated by AM symbiosis in the shoots under control conditions (Ri (control_shoot) column) (**Figure 5** and **Supplementary Table S4**). These results implied that under control conditions, AM symbiosis already had a similar effect as salinity on the expression of these genes. Similar results were also observed in the expression pattern of genes belonging to “ester-related” or “oxidoreductase activity, acting on CH-OH group of donors” GO terms (**Figure 6** and **Supplementary Table S5**) and those belonging to reproduction-related GO terms (**Supplementary Figure S4** and **Supplementary Table S6**). To closely investigate the expression pattern and validate RNA-seq data, the expression profile from RNA-seq data and the corresponding RT−qPCR results for two genes were represented. For *granule-bound starch synthase II (GBSSII)* (Baysal et al., 2020) identified from the “glucan biosynthetic process” GO term, its expression was downregulated by AM symbiosis under control conditions and by salinity under mock conditions in both shoots and roots. Therefore, its expression was similar in mock and mycorrhizal tissue under salt stress. The RT−qPCR results were consistent with the RNA-seq findings (**Supplementary Figure S5**). For *xyloglucan endotransglucosylase/hydrolases 19 (XTH19)* (Yokoyama et al., 2004) identified from the “cellular glucan metabolic process” GO term, salinity or AM symbiosis did not influence the expression in the roots. However, its expression was upregulated by AM symbiosis under control conditions and by salinity under mock conditions in the shoots. Therefore, its expression was similar in mock and mycorrhizal shoots under salt stress. The RT−qPCR results were consistent with the RNA-seq findings (**Supplementary Figure S5**).

### Phosphate-related pathways may play important roles in AM-enhanced salt tolerance

**Figure 7:**
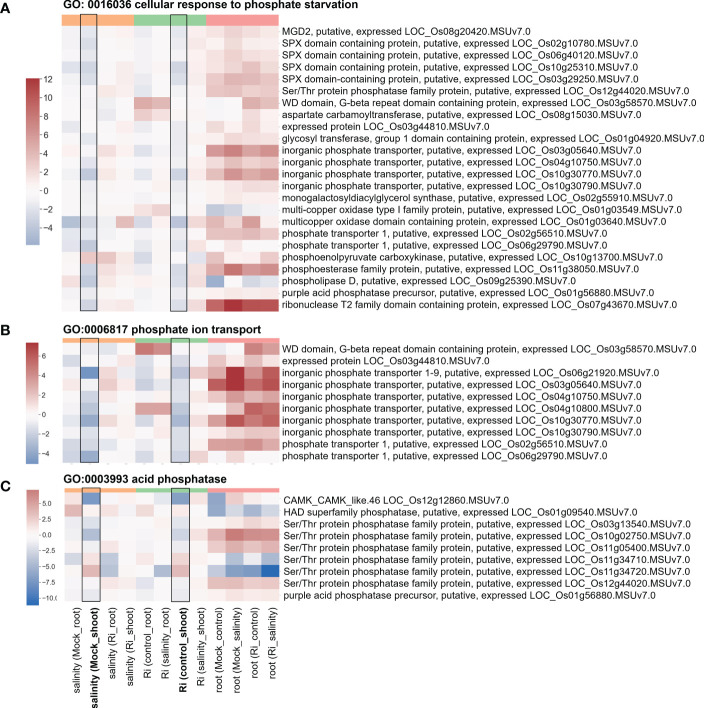
Heatmap showing the fold change of DEGs belonging to Pi homeostasis-related GO terms **(A)** Expression profile of DEGs from region 7 with the GO term “GO: 0016036 cellular response to phosphate starvation”. **(B)** Expression profile of DEGs from region 7 with the GO term “GO:0006817 phosphate ion transport”. **(C)** Expression profile of DEGs from region 7 with the GO term “GO:0003993 acid phosphatase”. The log2(fold change) values of DEGs from the selected GO terms were visualized with a heatmap. The color bar on top of the heatmap indicates the DE comparison groups: orange – salinity effect; green – AM symbiosis; pink – tissue difference. On the x-axis, each column represents a pairwise comparison to show the effect causing the DE, and inside the parentheses are the condition.

Several region 7-specific GO terms related to Pi homeostasis were selected for closer examination. For genes belonging to the GO terms “GO: 0016036 cellular response to phosphate starvation”, “GO:0006817 phosphate ion transport” and “GO:0003993 acid phosphatase”, most genes showed higher expression in the roots than in the shoots (**Figure 7** and **Supplementary Table S7**). Interestingly, similar to the genes mentioned above, the expression patterns of these genes in response to salinity and AM symbiosis were similar in the shoots. For example, SPX domain containing protein (LOC_Os10g25310) and Ser/Thr protein phosphatase family protein (LOC_Os11g34720) were repressed or induced by salinity in the mock shoots (salinity (Mock_shoot) column) and repressed or induced by AM symbiosis in the shoots under control conditions (Ri (control_shoot) column), respectively (**Figure 7** and **Supplementary Table S7**). These results implied that under nonsaline conditions, AM symbiosis influenced the expression of these genes in a similar way as salinity. Among these genes, the expression of *low phosphate root 5 (LPR5)* (Cao et al., 2016) identified from the “cellular response to phosphate starvation” GO term was downregulated by AM symbiosis under control conditions and by salinity under mock conditions in both shoots and roots. Therefore, its expression was similar in mock and mycorrhizal tissues under salt stress. The RT−qPCR results were consistent with the RNA-seq findings (**Supplementary Figure S5**). For *purple acid phosphatase 7 (PAP7)* (Zhang et al., 2011) identified from the “acid phosphatase” GO term, salinity or AM symbiosis did not influence its expression in the roots. However, its expression was upregulated by AM symbiosis under control conditions and by salinity under mock conditions in the shoots. Therefore, its expression was similar in mock and mycorrhizal shoots under salt stress. The RT−qPCR results were consistent with the RNA-seq findings (**Supplementary Figure S5**). Overall, these data implied that AM symbiosis influenced the expression of genes belonging to cell wall modification, ester-related, reproduction, oxidoreductase activity and phosphate-related pathways as salinity did, and this mediation might help plants prepare while encountering salinity stress.

### AM-regulated genes under salinity stress may be involved in AM-enhanced salt tolerance

To identify AM-regulated genes under salt stress, especially from the g3 region, the cutoff of log2(fold change) was set to one to obtain AM-up or downregulated genes under salt stress. AM-upregulated genes, especially in the roots under salinity, included AMP-binding enzyme, GRETCHEN HAGEN 3.2 (GH3.2), glucose-1-phosphate adenylyltransferase, inorganic phosphate transporter, multicopper oxidase and starch synthase. AM-downregulated genes, especially in the roots under salinity, included kinases such as calcium/calmodulin dependent protein kinase (CAMK) and receptor-like protein kinase; Ser/Thr protein phosphatase; and cell wall-related genes such as endoglucanase, expansin and pectinesterase (**Supplementary Figure S6** and **Table S8**). AM-upregulated genes, especially in the shoots under salinity, included cation/hydrogen exchanger 15 (CHX15); Pi-related genes, such as SPX domain-containing protein, inorganic phosphate transporter and multicopper oxidase; cell wall-related genes, such as endoglucanase, expansin and pectinesterase; inositol-3-phosphate synthase; and potassium channel Arabidopsis K^+^ transporters 2/3 (AKT2/3). Fewer genes were downregulated by AM symbiosis, especially in shoots under salt stress, and a high proportion of terpene synthase was identified from this category (**Supplementary Figure S7** and **Table S8**). Interestingly, AM symbiosis also had a similar effect as salinity on the expression of these genes under control conditions, especially in the shoots (**Supplementary Figures S6** and **S7** and **Table S8**).

## Discussion

We found that AM symbiosis did not affect shoot and root dry weight under nonsaline conditions (**Figure 1C**). Most importantly, we found that the shoot dry weight of rice colonized by *R. irregularis* was significantly higher than that of the mock rice under salt stress (**Figure 1C**), which is in accordance with previous studies in rice cv. Puntal colonized by *Claroideoglomus etunicatum* (Porcel et al., 2015; Porcel et al., 2016), and in *Solanum lycopersicum* colonized by *R. irregularis* (Hajiboland et al., 2010; Khalloufi et al., 2017). Thus, we proved that AM symbiosis could improve rice shoot growth under salt stress. In addition, we further proved that AM symbiosis could also enhance reproductive growth under salinity (**Figures 1D–F**).

Our mineral nutrient analysis showed that mycorrhizal plants colonized with *R. irregularis* exhibited lower shoot Na^+^ concentrations under salt stress, consistent with previous research in *Trigonella foenumgraecum* colonized by *R. irregularis* (Evelin et al., 2012) and in rice cv. Leum Pua (Indica rice) colonized by *Glomus. geosporum* or *G. mosseae* (Tisarum et al., 2020). However, the Na^+^ concentration in the shoot tissues of rice cv. Puntal (Indica rice) was not affected by the AM fungi *C. etunicatum* (Porcel et al., 2016). These results suggest that different combinations of rice cultivars, AM fungal species, and salinity treatment might result in distinct outcomes. Furthermore, the reduced root-to-shoot Na^+^ distribution in mycorrhizal plants under salt stress (**Supplementary Figure S1E**) was consistent with the finding that the AM fungus *C. etunicatum* reduced the root-to-shoot Na^+^ distribution in rice cv. Puntal (Porcel et al., 2016). Under salt stress, increased shoot K^+^/Na^+^ ratio by *R. irregularis* colonization was observed in rice (our study), *S. lycopersicum* (Hajiboland et al., 2010) and *T. foenumgraecum* (Evelin et al., 2012). Moreover, previous studies have shown that AM fungi (Ri) alleviate the stress impact by reducing ROS accumulation (Benhiba et al., 2015; He et al., 2017; Haddidi et al., 2020), which is consistent with our results (**Figure 2**).

Changes in plants’ ability to respond or tolerate salt stress may be achieved through regulation of gene expression. Surprisingly, our results showed that AM symbiosis influenced the expression of genes belonging to cell wall GO term in a similar way as salinity did, especially in the shoots under nonsaline conditions, and several expasin and pectinesterase were upregulated by AM symbiosis under salt stress in shoots (**Figure 5** and **Supplementary Figure S7**). Cell wall is the first organelle that senses and responds to salt stress (Evelin et al., 2019). Pectinesterase was identified as a promising candidate gene involved in salinity tolerance from an integrative meta-analysis approach in rice (Mansuri et al., 2020). Glycosyl hydrolase has been reported to be a potential biomarker for salinity tolerance in tomato varieties (Reyes-Pérez et al., 2019). Overexpression of expansin can enhance the tolerance of crop plants to salt stress (Han et al., 2012; Jadamba et al., 2020). Under salinity, lipid metabolism can be associated with extreme alterations in cell membrane integrity and function (Evelin et al., 2019). AM symbiosis upregulated the expression of most GDSL-like lipase/acylhydrolase belonging to ester-related GO terms in the shoots under nonsaline condition in a similar way as salinity did (**Figure 6**), and the positive role of AtLTL1 in salt tolerance has been reported (Naranjo et al., 2006). Myo-inositol and its derivative metabolites, such as inositol-3-phophate, are essential in diverse signal transduction responding to stress conditions (Jia et al., 2019). We found that AM symbiosis also had a similar effect as salinity on the expression of inositol-3-phosphate synthase in the shoots under control conditions. Moreover, the expression of this gene was upregulated by AM symbiosis under salt stress in the shoots (**Supplementary Figure S7**). Previous studied also showed that salt tolerance could be enhanced by the overexpression of inositol-3-phosphate synthase in rice (Kusuda et al., 2015). Phytohormones have been reported to play important roles in salt stress tolerance (Fahad et al., 2015). Our results found that AM symbiosis also had a similar effect as salinity on the expression of *GH3.2* under control conditions in the shoots. Moreover, the expression of this gene was upregulated by AM symbiosis under salt stress in the roots (**Supplementary Figures S6**). The GH3 protein family, which is responsible for binding free indole-3-acetic acid (IAA) to amino acids, has been reported to play a positive role in salt stress tolerance in cotton (Kirungu et al., 2019). Flavonoids could act as the nonenzymatic ROS scavenger (Yang and Guo, 2018), and 4CL proteins belonging to the AMP-binding protein family and regulating a pathway that contributes to flavonoid synthesis may contribute to salinity tolerance in two desert poplars (Zhang et al., 2015). Our data showed that AM symbiosis also had a similar effect as salinity on the expression of AMP-binding enzymes under control conditions in the shoots, and the expression of these genes was upregulated by AM symbiosis under salt stress in the roots (**Supplementary Figures S6**). Starch has been considered a key factor in plant fitness under abiotic stress, such as salinity (Kanai et al., 2007; Wang et al., 2013; Thalmann and Santelia, 2017). For example, the expression of glucose-1-phosphate adenylyltransferase, which encodes a starch building enzyme, was upregulated by salt stress in *Neochloris oleoabundans* (De Jaeger et al., 2018). The expression of starch synthase was also upregulated by salt stress in different plant species (Thalmann and Santelia, 2017). We found that AM symbiosis upregulated the expression of starch synthase and glucose-1-phosphate adenylyltransferase in the roots under salt stress (**Figure 5** and **Supplementary Figure S6**), which could be beneficial for salinity tolerance. However, AM symbiosis downregulated the expression of these genes in the shoots under nonsaline condition similar as the effect caused by salinity (**Figure 5** and **Supplementary Figure S6**). Reduced starch synthesis or increased starch remobilization might be helpful to provide sugars as osmoprotectants to mitigate the negative effect of stress (Thalmann and Santelia, 2017).

Maintain ionic homeostasis is important for plant salt stress tolerance (Yang and Guo, 2018; Evelin et al., 2019). “Cytoplasmic membrane-bounded vesicle” is the most enriched GO term among genes showed tissue-preferred expression in response to both AM symbiosis and salinity (**Supplementary Table S2**). It has been shown that intracellular membrane dynamics have played roles in plant salt tolerance. Under salt stress, regulation of vesicle trafficking and increase of cytoplasmic vesicles by accelerating endocytosis may be helpful for Na^+^ compartmentalization (Yang and Guo, 2018; Wang et al., 2020). Our results also showed that AM symbiosis and salinity regulated genes involved in Pi homeostasis in a similar way in shoots, and AM symbiosis upregulated the expression of genes such as inorganic Pi transporter and multicopper oxidase under salinity in both roots and shoots (**Figure 7** and **Supplementary Figures S6**, **7**). Salinity-reduced Pi uptake has been reported in several plant species, suggesting the negative role of salt stress on Pi uptake (Martinez and Lauchli, 1994; Navarro et al., 2001). Many genes involved in the Pi starvation response are also regulated by salinity or play important roles in salt stress tolerance, suggesting crosstalk between the Pi starvation response and the salt stress signaling pathway (Baek et al., 2017). These findings support that many Pi homeostasis-related genes were regulated by salinity in our transcriptome analysis. In addition, previous studies showed that salt stress tolerance could be enhanced by exogenous Pi application or in Pi-accumulating mutants (Okusanya and Fawole, 1985; Miura et al., 2011; Sun et al., 2018). The increased Pi content in mycorrhizal roots and shoots and upregulated expression of Pi homeostasis-related genes by AM symbiosis observed in our study might be the key factor for AM-enhanced salt stress tolerance (**Supplementary Figures S1**, **S6**, **S7**). AM symbiosis also had a similar effect as salinity on the expression of cation/H^+^ exchanger (CHX) and Arabidopsis K^+^ transporter (AKT2/3) in the shoots under control conditions, and AM-upregulated expression of these two genes was observed in the shoots under salt stress (**Supplementary Figures S7**). The contribution of the CHX channel to salt stress tolerance has been reported in soybean (Jia et al., 2017). AKT2 channels play a major role in phloem K^+^ loading and unloading, and the fact that AKT2 is regulated by two positive regulators of salt stress tolerance, calcineurin B-like-interacting protein kinase 6 (CIPK6) and calcineurin B-like 4 (CBL4), suggests that AKT2 is involved in the adaptation to salt stress (Chérel and Gaillard, 2019).

Overall, our results indicate that AM symbiosis might possibly use a multipronged approach to influence gene expression in a way similar to salinity did, especially in the shoots under nonsaline conditions. This modification might help plants be prepared for salt stress. However, the underlying molecular mechanism to mediate this regulation is still unclear, and the relevance of this regulation on salt stress tolerance is also unknown. In addition, under salt stress, AM symbiosis also upregulated the expression of several genes involved in cell wall and lipid modification, inositol-3-phophate and starch synthesis, and auxin and ionic homeostasis. These genes may play positive roles in salt stress tolerance and the growth of mycorrhizal plants might be further maintained under salinity.

## Data availability statement

The datasets presented in this study can be found in online repositories. The names of the repository/repositories and accession number(s) can be found in the article/**Supplementary material**.

## Author contributions

Y-HC and S-YY designed the experiments. Y-HC and K-CC performed the experiments and analyzed the data. CH performed the RNA sequencing analysis. CH, Y-HC and S-YY wrote the article. All authors read and approved the final manuscript. All authors contributed to the article and approved the submitted version.
