# Supplementary material for: Transcriptome analysis reveals the mechanisms for mycorrhiza-enhanced salt tolerance in rice
Source: Front Plant Sci. 2022 Dec 19;13:1072171. doi: 10.3389/fpls.2022.1072171 (PMC9806932; doi:10.3389/fpls.2022.1072171)
Supplement: Supplementary file 1 [file DataSheet_1.pdf]

FITURE S1

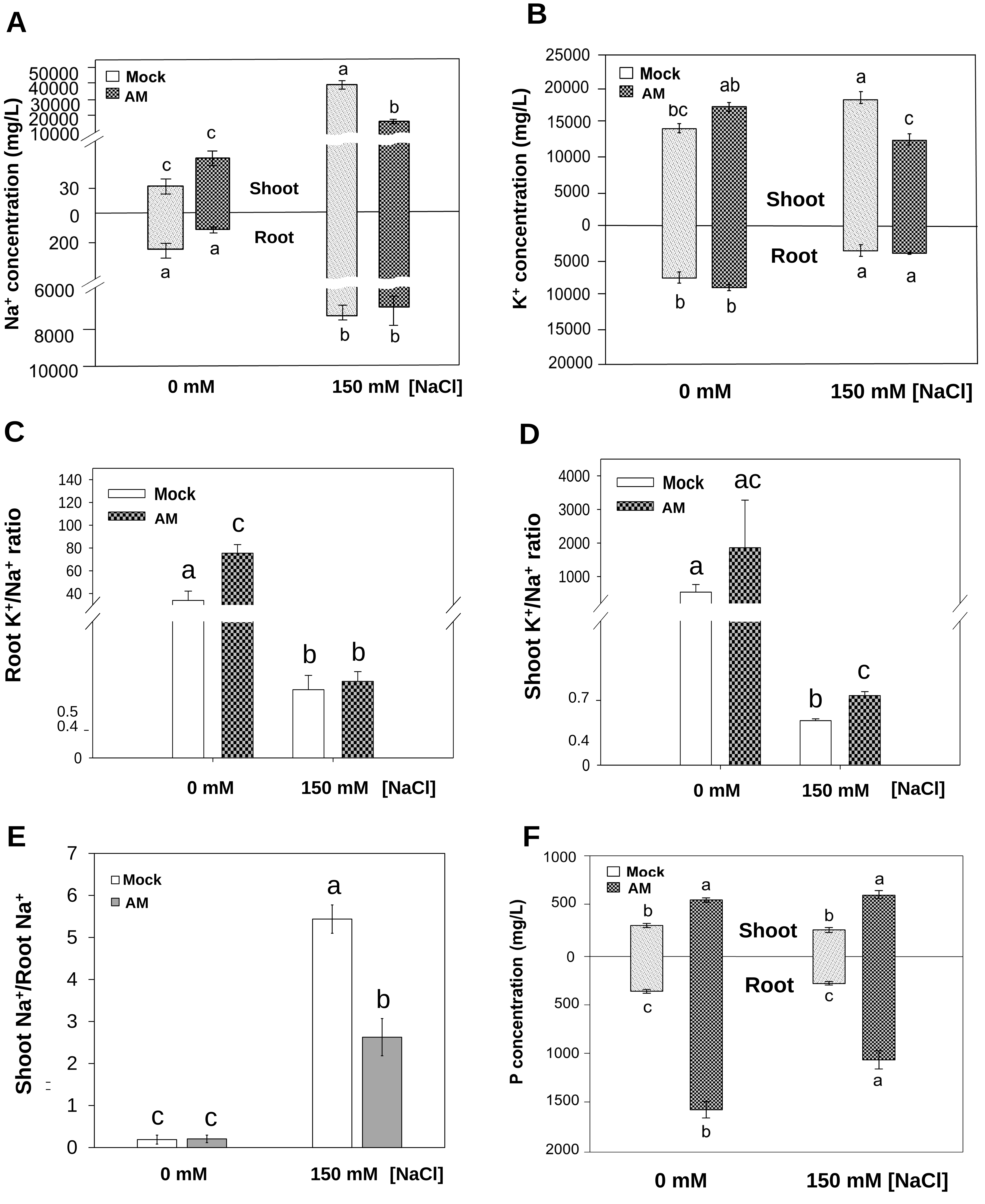

FIUGRE S1 Effects of salinity and fungal inoculation on ion concentration in rice plants under salt stress.

(A) Na^+ concentrations. **(B)** K^+ concentration. **(C)** K^+/Na^+ ratio of roots. **(D)** K^+/Na^+ ratio of shoots. **(E)** Shoot Na^+/root Na^+ ratio of plants. **(F)** Phosphorus (P) concentration. Rice plants were grown without (mock) or with AM fungi *R. irregularis* (AM) for 5 weeks and then treated with normal (0 mM NaCl) or salt solution (150 mM NaCl) for 3 weeks. The ion concentration was measured by ICP-OES. Standard error is derived from 3 biological replicates (3 plants were considered as one biological replicate). Shoot and root samples were analyzed separately in (A), (B) and (F). Different letters represent significant differences at $p < 0.05$ (one-way ANOVA followed by a least significant differences post hoc test) in (A)(B)(E)(F); $p < 0.1$, pairwise t test in (C) (D).

FIGURE S2

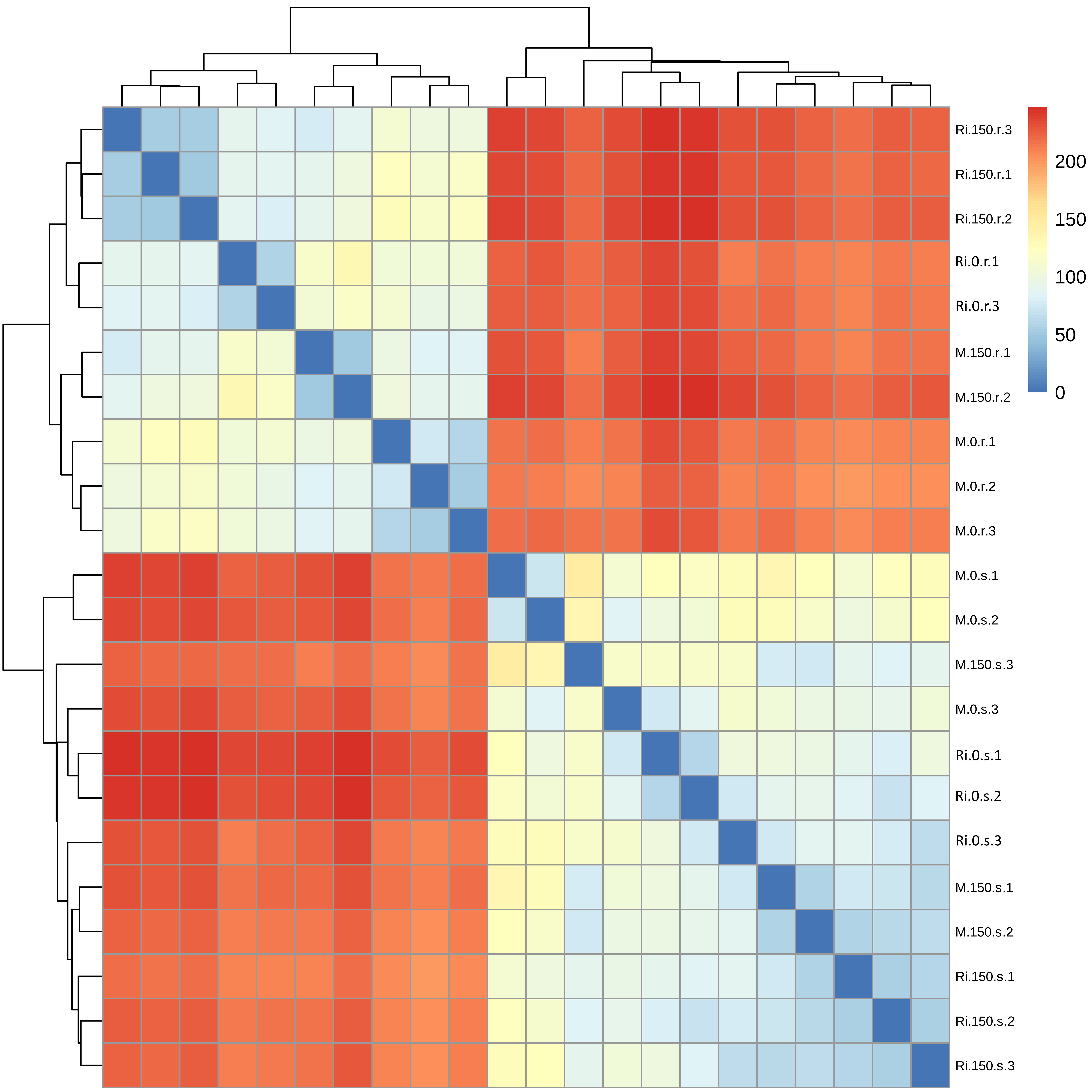

FIGURE S2. Heatmap of Pearson correlations between biological replicates
The total reads counts were normalized using the VST method from DESeq2. M, Mock; Ri, AM fungi *R. irregularis*; r, root; s, shoot; 0, 0 mM; 150, 150 mM.

FIGURE S3

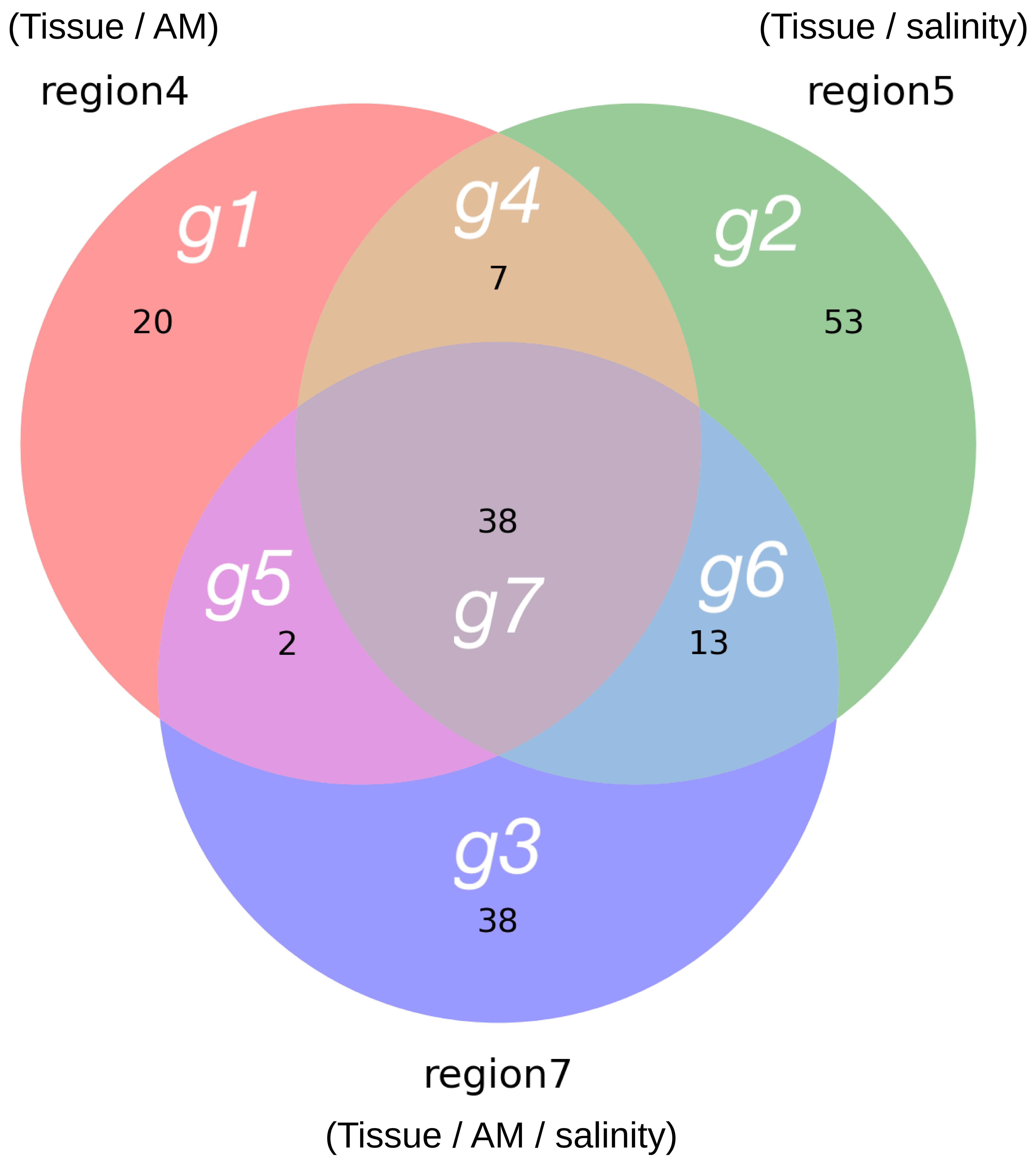

Figure S3 Venn diagram of GO terms from the region of interest: region 4, region 5, region 7.
GO terms were identified from the DEGs in the region 4, region 5, region 7 according to genome annotation from Phytozome. White labels are the name for each GO region. Black numbers are the number of GO terms in each GO region.

FIGURE S4

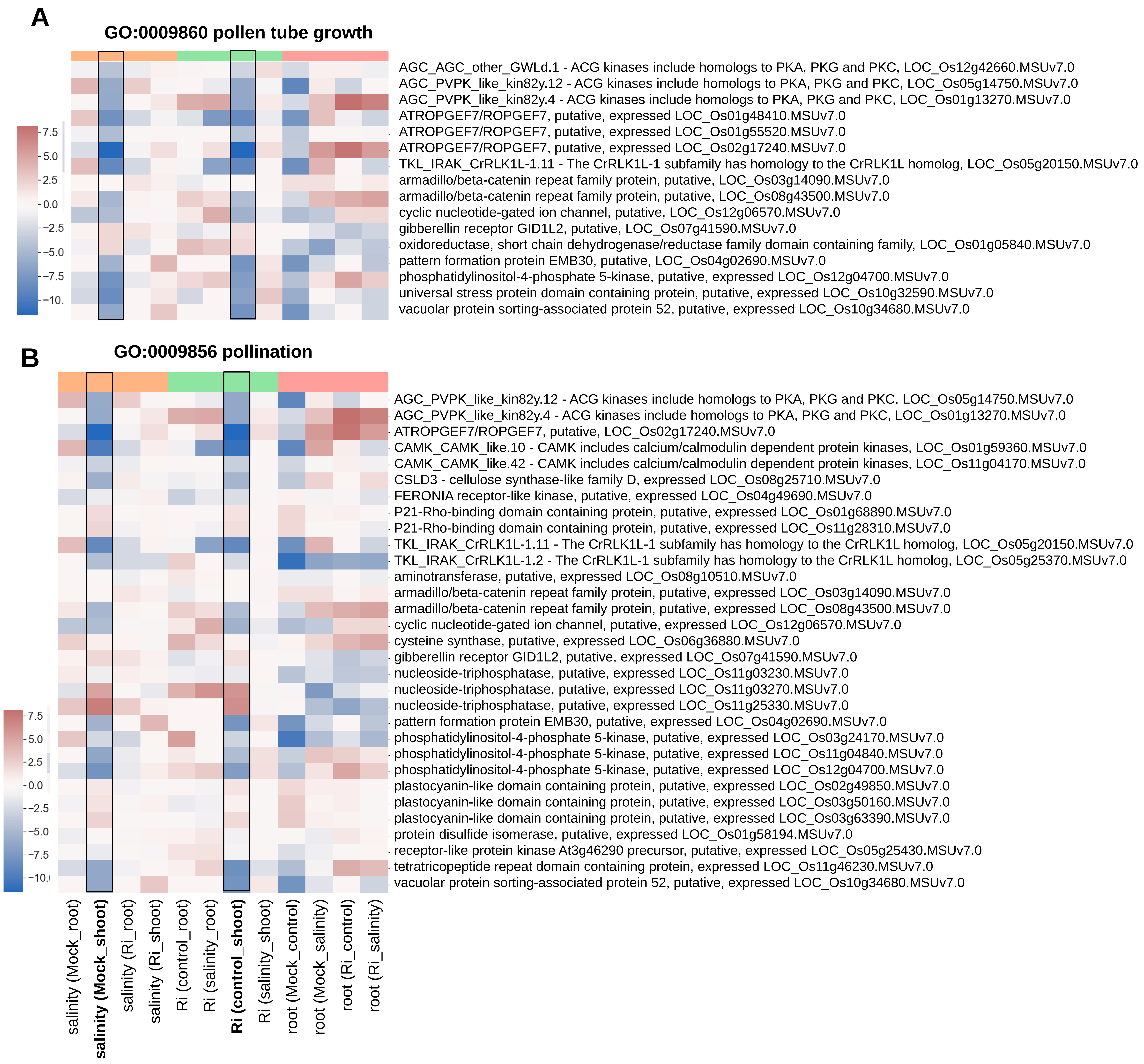

Figure S4 Heatmap showing the fold change of DEGs belonging to reproduction-related GO terms. (A) Expression profile of DEGs from region 7 with the GO term "GO:0009860 pollen tube growth". **(B)** Expression profile of DEGs from region 7 with the GO term "GO:0009856 pollination ". The log2(fold change) values of DEGs from the selected GO terms were visualized with a heatmap. The color bar on top of the heatmap indicates the DE comparison groups: orange – salinity effect; green – AM symbiosis; pink – tissue difference. On the x-axis, each column represents a pairwise comparison to show the effect causing the DE, and inside the parentheses are the condition.

FIGURE S5

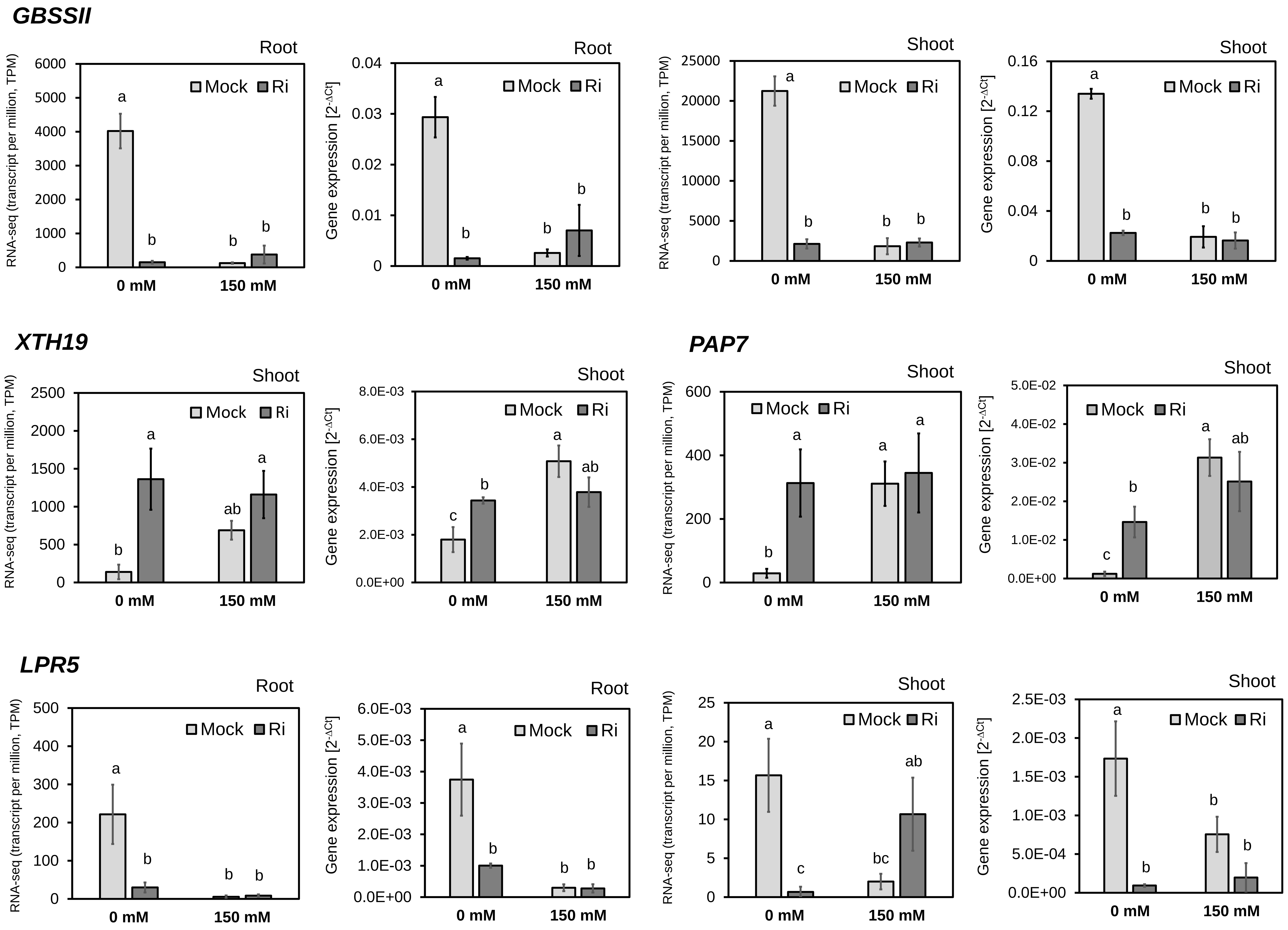

FIGURE S5 Quantitative reverse transcription PCR (RT–qPCR) validation of the DEGs identified from RNA-seq analysis.
Transcript per million fragments (TPM) values and the expression level measured by RT-qPCR analysis were shown. Rice plants were grown without (mock) or with AM fungi *R. irregularis* (Ri) for 5 weeks and then treated with normal (0 mM NaCl) or salt solution (150 mM NaCl) for 3 weeks. Gene expression was normalized with *Cyclophilin2*. Standard error is derived from 3-6 biological replicates (2 plants were considered as one biological replicate). Two-way ANOVA followed by a least significant differences post hoc test, $p<0.1$.

FIGURE S6

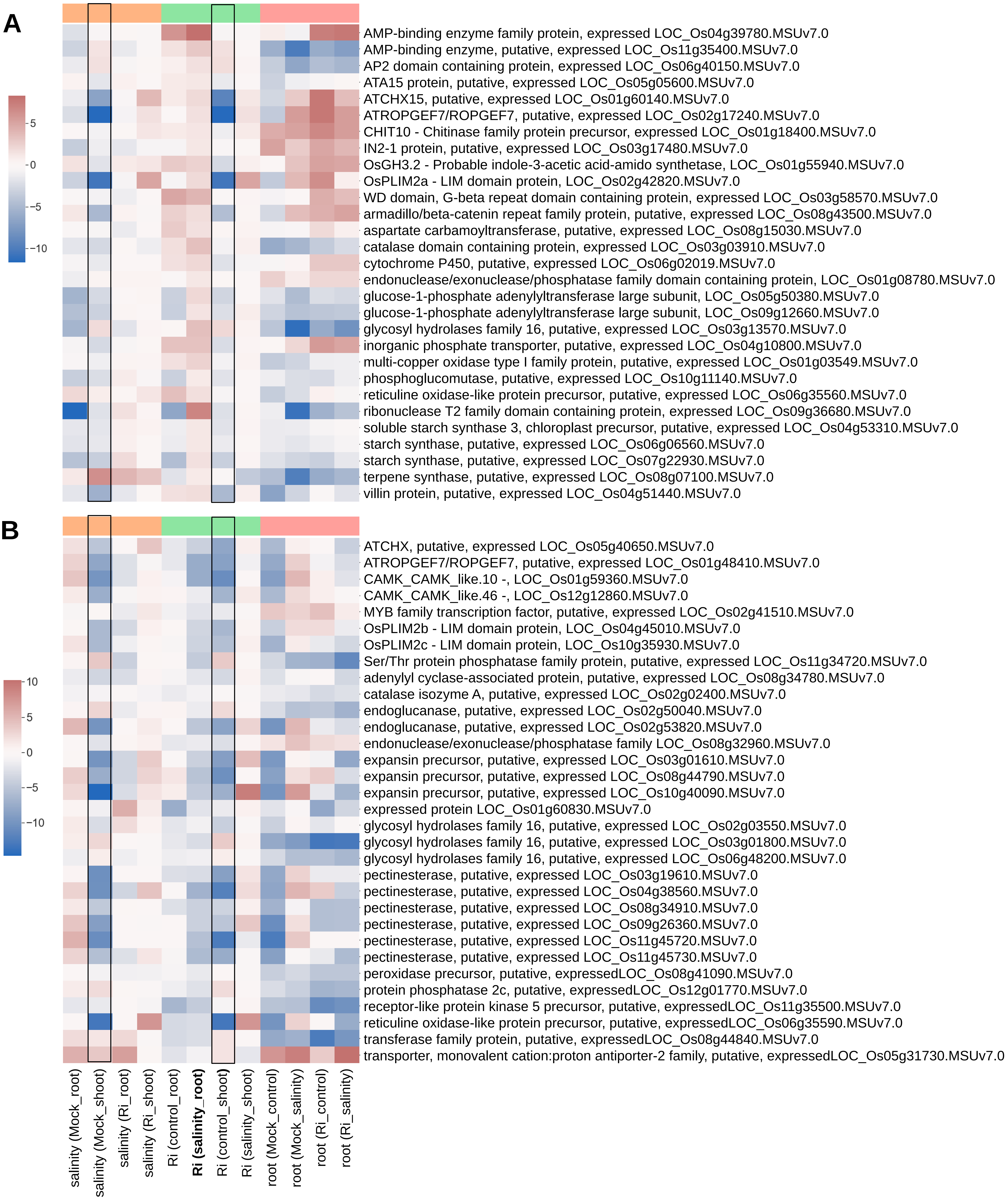

Figure S6. Heatmap showing the fold change of AM-regulated DEGs under salinity in roots
(A) AM-upregulated DEGs. **(B)** AM-downregulated DEGs. The log2(fold change) values of AM-regulated DEGs were visualized with a heatmap. The color bar on top of the heatmap indicates the DE comparison groups: orange – salinity effect; green – AM symbiosis; pink – tissue difference. On the x-axis, each column represents a pairwise comparison to show the effect causing the DE, and inside the parentheses are the condition.

FIGURE S7

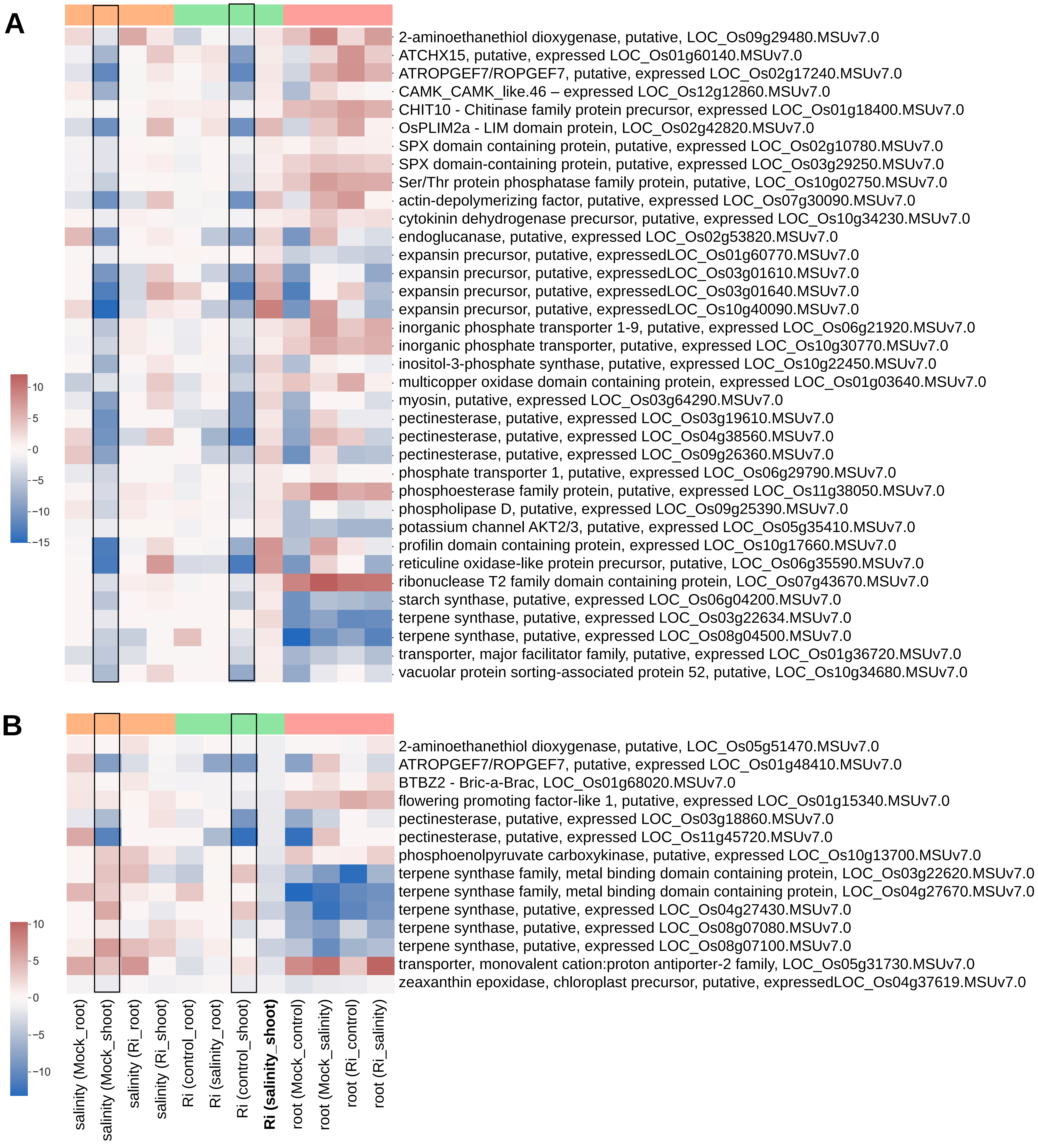

Figure S7. Heatmap showing the fold change of AM-regulated DEGs under salinity in shoots (A) AM-upregulated DEGs. **(B)** AM-downregulated DEGs. The log2(Fold Change) value of AM-regulated DEGs were visualized with heatmap. The color bar on top of the heatmap indicated the DE comparison groups: orange – salinity effect; green – AM symbiosis; pink – tissue difference. On the x-axis, each column represents a pairwise comparison to show the effect causing the DE, and inside the parentheses are the condition.
